# Quantitative Characterization of Glycan-Receptor Binding of H9N2 Influenza A Virus Hemagglutinin

**DOI:** 10.1371/journal.pone.0059550

**Published:** 2013-04-23

**Authors:** Karunya Srinivasan, Rahul Raman, Akila Jayaraman, Karthik Viswanathan, Ram Sasisekharan

**Affiliations:** Harvard-MIT Division of Health Sciences and Technology, Koch Institute for Integrative Cancer Research, Singapore-MIT Alliance for Research and Technology, Department of Biological Engineering, Massachusetts Institute of Technology (MIT), Cambridge, Massachusetts, United States of America; Centro de Biología Molecular Severo Ochoa (CSIC-UAM), Spain

## Abstract

Avian influenza subtypes such as H5, H7 and H9 are yet to adapt to the human host so as to establish airborne transmission between humans. However, lab-generated reassorted viruses possessing hemagglutinin (HA) and neuraminidase (NA) genes from an avian H9 isolate and other genes from a human-adapted (H3 or H1) subtype acquired two amino acid changes in HA and a single amino acid change in NA that confer respiratory droplet transmission in ferrets. We previously demonstrated for human-adapted H1, H2 and H3 subtypes that quantitative binding affinity of their HA to α2→6 sialylated glycan receptors correlates with respiratory droplet transmissibility of the virus in ferrets. Such a relationship remains to be established for H9 HA. In this study, we performed a quantitative biochemical characterization of glycan receptor binding properties of wild-type and mutant forms of representative H9 HAs that were previously used in context of reassorted viruses in ferret transmission studies. We demonstrate here that distinct molecular interactions in the glycan receptor-binding site of different H9 HAs affect the glycan-binding specificity and affinity. Further we show that α2→6 glycan receptor-binding affinity of a mutant H9 HA carrying Thr-189→Ala amino acid change correlates with the respiratory droplet transmission in ferrets conferred by this change. Our findings contribute to a framework for monitoring the evolution of H9 HA by understanding effects of molecular changes in HA on glycan receptor-binding properties.

## Introduction

Among subtypes of influenza A viruses isolated from avian species, H5N1, H7N2, H7N3, H7N7 and H9N2 have been known to infect humans but are yet to adapt to human host so as to establish airborne human-to-human transmission. In the past few years, novel influenza strains such as 2009 H1N1 and 2010 H3N2 that naturally emerged from multiple reassortment of viral gene segments between avian, swine and human isolates were able to successfully adapt to human host [1], [2]. In the context of these novel strains, the avian influenza subtypes pose a significant threat of human adaptation [3]. With the human population predominantly naïve to these avian influenza antigens, constant surveillance with particular focus on molecular changes geared towards human host adaptation becomes vital in this era of pandemics [4].

The adaptation of influenza A viruses to human host has been studied extensively in model animal systems such as ferrets [5], [6]. One of the important factors governing human adaptation of the virus is the gain in its ability to transmit via respiratory droplets in the ferret animal model [5], [6]. The transmissibility of human-adapted pandemic influenza strains, 1918 H1N1, 1957 H2N2 and 2009 H1N1, in ferrets has been demonstrated to correlate with the specificity and quantitative affinity of the viral surface glycoprotein hemagglutinin (HA) binding to α2→6 sialylated glycans (or *human receptors*) [7]–[9]. These human receptors are predominantly expressed in the respiratory tract epithelium of humans and ferrets [10]–[12]. The HA of influenza viruses isolated from avian species typically binds to α2→3 sialylated glycans (or *avian receptors*) [11]. Therefore, the gain in the ability of HA from an avian isolate (such as H5, H7, H9, etc.) to preferentially bind to human receptors (high relative binding affinity to human receptor over avian receptor) is implicated as one of the important factors for the human adaptation of the virus [13].

H9 is unique among avian subtypes since its HA has naturally acquired a mutation in the 226 position (based on H3 numbering) in glycan-receptor binding site (RBS) from Gln to Leu [14]. Leu-226 is predominantly found in human-adapted H2 and H3 HAs and is critically involved in their binding to human receptors. It was demonstrated that among different avian H9N2 isolates, those with Leu-226 in the RBS showed a similar tropism of preferentially infecting non-ciliated human airway epithelial cells (characteristic of human-adapted viruses) [14]. Although the H9N2 virus is yet to adapt to a human host, reassorted viruses carrying HA and NA from a H9N2 virus isolated from a wild terrestrial bird (A/Guinea Fowl/Hong Kong/WF10/99 or WF10) and other genes from human-adapted H3N2 [15] or 2009 pandemic H1N1 [16] have been able to transmit via respiratory droplet in ferrets by acquiring as few as two amino acid changes in the HA, Thr-189→Ala in the RBS and Gly-192→Arg in the HA2 subunit.

The glycan receptor-binding properties of both H9N2 viruses isolated from avian species and reassorted viruses comprising of wild-type and mutant forms of H9 HA have been studied by screening them on glycan array platforms [17]. Such screening analyses served as a quick readout for the number of different types of α2→3 and α2→6 sialylated glycans that bind to the virus at a fixed high viral titer and limited biochemical information on glycan affinity and specificity. Previously, we demonstrated that correlating glycan-receptor binding properties from such screening assays to transmissibility of virus has major limitations. For example, two different H1N1 strains isolated from humans that showed binding to both α2→3 and α2→6 sialylated glycans in screening assays showed dramatically different transmissibility in ferrets [18]. However we demonstrated that HA from strain that transmitted efficiently quantitatively bound to human receptors with significantly higher affinity than HA from strain that showed inefficient transmission in ferrets [9]. Such a correlation remains to be determined for the H9 subtype in the context of the reassorted viruses that show respiratory droplet transmission in ferrets.

In this study, we focused on investigating the glycan receptor-binding specificity and affinity of H9 HAs from the representative avian isolates WF10 and A/Quail/Hong Kong/A28945/88 (Qa88) that had been recombinantly constructed via reverse genetics for ferret transmissibility studies carried out previously [15]–[17]. We first quantified glycan-receptor binding specificity of WF10 and Qa88 HAs using a dose-dependent glycan array assay mentioned above [9]. We then constructed homology-based structural models of these HAs to investigate the effect of these mutations on the glycan-receptor binding of HA. We finally made these mutations on WF10 and Qa88 HAs and experimentally quantified the glycan receptor-binding affinities of these mutant HAs. Our results demonstrated and corroborated that the mutations on H9 HAs that were found to confer respiratory droplet transmission in the ferret model also substantially increased human receptor-binding specificity and affinity in comparison to those of wild-type HAs.

## Results

As stated earlier, WF10 and Qa88 H9 HAs were chosen as representative HAs for characterization of glycan-receptor binding properties. These HAs are isolated from different avian hosts. While WF10 is from guinea fowl (wild terrestrial bird), Qa88 is from a strain that has been established in domestic poultry in China. WF10 HA has Leu-226 in the RBS while Qa88 has Gln-226 [14]. As pointed our earlier, reassorted viruses comprising of WF10 HA acquired additional mutations in the HA that conferred the viruses with the ability to transmit via respiratory droplets in ferrets after repeated passaging in these animals [15].

### Characterization of glycan receptor-binding properties of WF10 and Qa88 HAs

**Figure 1:**
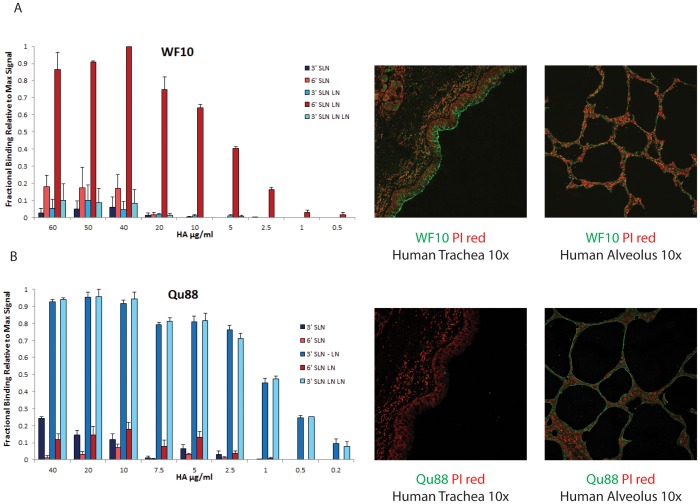
Glycan receptor-binding properties of WF10 and Qa88 HA. **A**, Dose-dependent direct binding of WF10 HA to glycan array (*left*) shows that it binds specifically to a representative human receptor (6′SLN-LN). Tissue staining (*right*) shows extensive staining of apical surface of human tracheal epithelia and minimal observable staining of alveolar tissue section by WF10 HA (in green) shown against propidium iodide staining (in red). **B**, Dose-dependent direct glycan array binding of Qa88 HA (*left*) shows specific binding to avian receptors (3′SLN-LN and 3′SLN-LN-LN). The panel on the right shows poor staining of apical surface of human tracheal epithelia and extensive staining of alveolar tissue section by Qa88 HA (in green) shown against propidium iodide staining (in red).

We previously developed a dose-dependent glycan array binding assay to quantitatively characterize glycan receptor binding affinity of HA by calculating an apparent binding constant Kd' [9], [19]. WF10 HA was recombinantly expressed and analyzed using this assay. WF10 HA showed a highly specific binding to a representative human receptor, a poly-lactosamine oligosaccharide terminated by α2→6 linked sialic acid (6′SLN-LN; see **Table S1** for details on glycan structure) (**Figure 1A**). Although the WF10 virus showed binding to both avian and human receptors in previous glycan array screening studies [17], our results indicate that its quantitative binding affinity to human receptor is orders of magnitude higher than that to avian receptor.

The Kd'∼300 pM for WF10 HA binding to 6′SLN-LN is 5 fold higher than that of 2009 H1N1 HA (Kd'∼1.5 nM) [7] and 60-fold lower than that of 1918 H1N1 and 1958 H2N2 (Kd' ∼5 pM) HA [8], [9]. The human receptor-binding property of WF10 based on the glycan array was consistent with its extensive staining of apical surface of human tracheal epithelium (**Figure 1B**). As we have demonstrated earlier [19], the apical surface of the human tracheal section comprises of both ciliated cells and non-ciliated (goblet) cells, which predominantly express human receptors. In the context of the human tracheal tissue section, WF10 binds to both these cells and this binding pattern is consistent with what we have observed previously for HA from prototypic human-adapted viruses [8], [9].

Qa88 HA, on the other hand showed predominant binding (Kd'∼30 pM) to avian receptors, polylactosamine oligosaccharides terminated by α2→3-linked sialic acid (3′SLN-LN and 3′SLN-LN-LN; see **Table S1** for details on glycan structure). Qa88 HA showed minimal binding to human receptors (**Figure 1C**). Therefore not only does the Qa88 virus preferentially bind to higher number of avian receptors as observed in previous glycan array screening studies [17], the quantitative avian receptor-binding affinity of Qa88 is orders of magnitude higher than its human receptor-binding affinity. Furthermore, the avian receptor-binding property of Qa88 HA was consistent with its extensive staining of human alveolar tissue section (**Figure 1D**), which predominantly expresses these receptors [11].

### Molecular Interactions of WF10 and Qa88 HA with avian and human receptors

**Figure 2:**
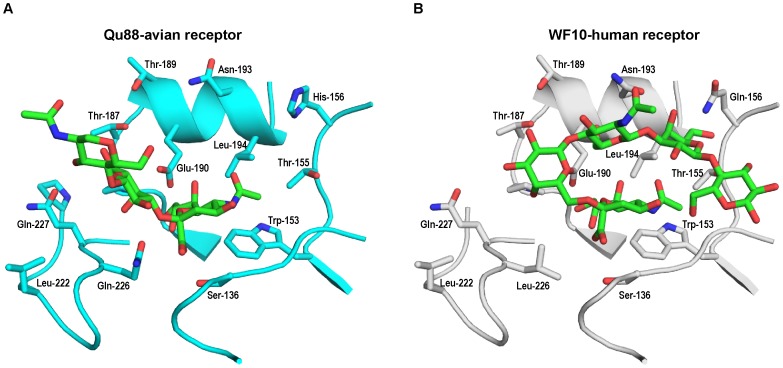
Structural model of H9 HA-glycan receptor complexes. A, Structural model of Qa88 HA with an avian receptor. The glycan receptor-binding site of HA is shown as a cartoon (carbon atom colored in *cyan*) with side chains of key residues in the RBS shown in stick representation. B, Structural model of WF10 with human receptor. The glycan receptor-binding site of HA is shown as a cartoon (carbon atom colored in *gray*) with side chains of key residues in the RBS shown in stick representation. The glycan receptor is shown in stick representation (carbon atom colored in *green*)

To better understand the contrasting glycan-binding properties of WF10 and Qa88 HAs we analyzed the molecular interactions of these HAs with representative avian and human receptors. The X-ray crystal structures of a swine H9 HA and its complexes with an avian (LS-Tetrasaccharide a; LSTa – see **Table S1** for details on glycan structure) and human receptor (LS-Tetrasaccharide c; LSTc - – see **Table S1** for details on glycan structure) [20] were used to generate structural models of Qa88-LSTa and WF10-LSTc complexes (**Figure 2**). Analysis of these structural complexes shows differences in amino acids that constitute the glycan receptor-binding site (RBS) of WF10 and Qa88 HAs. Specifically, there are differences in residues at positions 156 and 226 (numbering is based on H3 HA) and also orientation of side chain of Asn-193. There is also a difference in amino acid at the 137 position (which is not shown in the figure for the sake of clarity) wherein WF10 has Arg while Qa88 has Lys at this position.

In the case of RBS of Qa88 HA, Gln-226 is positioned to make ionic contact with glycosidic oxygen atom of Neu5Acα2→3Gal linkage. On the other hand, in WF10, Leu-226 is positioned to make van der Waals contact with C-6 atom of Neu5Acα2→6Gal linkage. Leu-226 is also one of the hallmark amino acid changes associated with switch to human receptor preference in H2 and H3 HA. In the case of H3 HA, Gly-228→Ser is another hallmark amino acid change. Based on the conformation of the LSTc in binding site of WF10 and the amino acid contacts in WF10 RBS made with this human receptor, it appears that amino acid at 228 position may not be as critical (as in the case of H3 HA) in governing quantitative human receptor binding preference of WF10 HA. The difference in nature of contacts involving amino acid position 226 is one of the molecular features that explain the differences in the glycan receptor-binding specificity of WF10 and Qa88 HAs.

The residue at 156 position appear to be involved in making contacts with the human receptor and not the avian receptor and therefore changes in this position is likely to affect binding of HA to human receptors. The orientation of side chain of Asn-193 is such that it is positioned to make contact with human receptor (in WF10-LSTc complex) and not the avian receptor (in Qa88-LSTa complex). There appear to be interconnected networks of inter-residue interactions involving the following sets of residue positions, {136, 137, 226}, {186, 222, 227}, {183,186, 187, 189, 190} and {187, 189, 193, 156}. These interaction networks are likely to govern the orientation of the side chains of the residues at the corresponding positions in the network, which in turn would govern contacts with glycan receptor. Therefore, differences in the amino acids at positions 137, 156, 183, 186, 187, 189, 193, 222, 226, and 227 between different H9 HAs would impinge on the quantitative glycan receptor-binding specificity of the HAs.

One of the observed mutations in WF10 HA in the reassorted virus that shows airborne transmission in ferrets is Thr-189→Ala. Although Thr-189 does not make direct contacts with both avian and human receptor, this position is a part of two critical amino acid interaction networks involving key residues at positions 190, 193 and 156 that are involved in making contact with the glycan receptor. Therefore, we postulated that the Thr-189→Ala mutation, in the context of a given HA, would alter binding to both avian and human receptors by affecting side chain orientation of residues (in a given HA) that make contact with these receptors.

### Design of WF10 and Qa88 HA mutants and characterization of their glycan receptor-binding properties

To experimentally test the effect of changes in the amino acid positions (based on the above structural analysis) on the glycan receptor-binding properties of WF10 and Qa88 H9 HAs, we designed the following mutant forms of these HAs. We designed two mutant forms of WF10 HA; mWF10:T189A (to investigate the effect of Thr-189→Ala mutation on human receptor-binding) and mWF10:L226Q (to determine if Leu-226→Gln mutation would change its binding preference from human to avian receptor). In the case of Qa88 HA, we defined three mutant forms; mQa88:T189A (to establish if changes to Thr-189 would affect avian receptor-binding), mQa88:Q226L (to determine if Gln-226→Leu mutation would change its binding preference from avian to human receptor), and mQa88:Q226L/T189A (to explore if the additional Thr189→Ala mutation would further modulate human receptor-binding of mQa88:Q226L). The amino acid changes were made to WF10 and Qa88 using site-directed mutagenesis and the glycan-receptor binding properties of the mutant HAs were characterized using glycan array and human tissue binding analyses.

**Figure 3:**
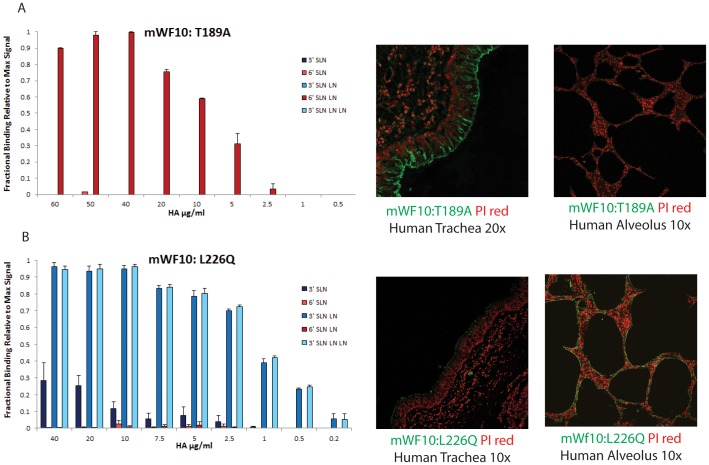
Glycan receptor-binding properties of mWF10:T189A and mWF10:L226Q. **A**, Dose-dependent direct glycan array binding of mWF10:T189A HA (*left*) shows increased specificity to the human receptor 6′SLN-LN when compared to WF10 (**Figure 1A**, left panel). Tissue-binding of mWF10:T189A HA (*right*) shows extensive staining of apical surface of human tracheal section and minimal staining of human alveolar section mWF10: T189A HA in *green* against propidium iodide in *red*). **B**, Dose-dependent direct glycan array binding of mWF10:L226Q HA (*left*) shows a complete reversal in binding from human (6′ SLN-LN) to avian receptors (3′SLN-LN and 3′SLN-LN-LN). Tissue staining (*right*) shows poor staining of apical surface of human tracheal epithelia and extensive staining of human alveolar sections by mWF10:L226Q HA (in green) shown against propidium iodide staining (in red).

mWF10:T189A showed a more specific binding to 6′SLN-LN than WF10 HA where binding to other representative glycan receptors at >40 µg/ml concentration was minimal when compared to WF10 HA at the same concentration (**Figure 3A**). However the Kd' for the 6′SLN-LN binding of the mutant was the same as that of WF10 HA. Staining of mWF10:T189A HA on human tracheal tissue sections revealed a pattern similar to that observed with WT WF10 with extensive binding of the protein to the apical side and weak to no binding to the human deep lung alveolar tissue (**Figure 3A**). The single amino acid change in mWF10:L226Q mutant, on the other hand, completely reversed its glycan-binding preference from human to avian receptors (with Kd' ∼30 pM for 3′SLN-LN and 3′SLN-LN-LN) (**Figure 3B**). Tissue binding assay was consistent with the observed glycan specificity with no tracheal tissue staining observed and extensive alveolar tissue binding (**Figure 3B**).

**Figure 4:**
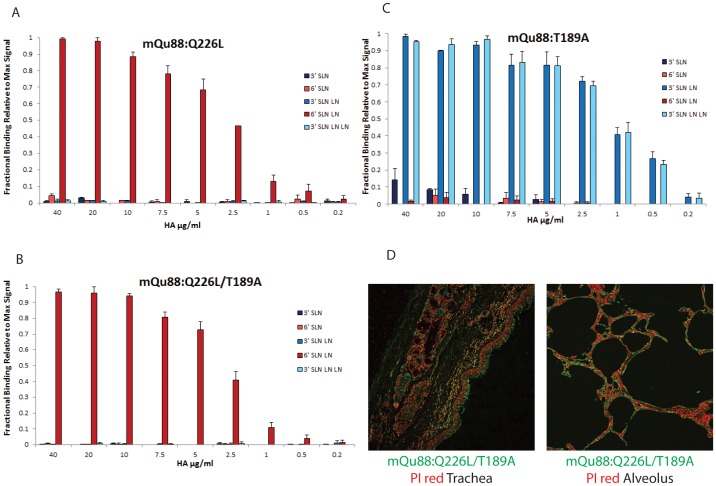
Glycan receptor-binding specificity of mQa88:Q226L, mQa88:T189A and double mutant mQa88:Q226L/T189A HA. **A**, shows dose-dependent direct glycan array binding of mQa88:Q226L. The single Q226L mutation shows binding exclusively to human receptors (6′ SLN-LN) on the array. **B**, shows dose-dependent direct glycan array binding of mQa88:Q226L/T189A. The additional T189A mutation increases binding specificity for human receptor (6′SLN-LN). **C**, Dose-dependent direct binding of mQa88:T189A shows that this mutant retains avian receptor-binding but specificity for avian receptors is higher when compared to mQa88 HA in Figure 1B. **D**, Consistent with glycan array-binding mQa88:Q226L/T189A HA shows extensive staining of apical surface of human tracheal epithelium and mQa88:T189A HA shows extensive staining of human alveolar section. HA (in green) shown against propidium iodide staining (in red).

In the case of Qa88 HA, a single amino acid change in the mQa88:Q226L mutant completely changed its glycan-binding preference from avian to human receptors (6′SLN-LN) (**Figure 4A**). Furthermore, the Kd' ∼50 pM for 6′SLN-LN binding of this mutant HA indicates that it shows a 5-fold increase in binding affinity to human receptor relative to the WF10 HA (which as Leu at 226 position). In fact the Kd' of mQa88:Q226L is in the same range as that of HAs from seasonal influenza strains [9]. Notably, both WF10 and mQa88:Q226L viruses showed similar pattern of binding to both avian and human receptors in the glycan array screening assays performed earlier [17]. Therefore, the glycan array screening of these viruses at single high titer was unable to capture these key differences and nuances in their quantitative human receptor-binding affinity.

The additional Thr-189→Ala change in the mQa88:Q226L/T189A mutant leads to an increase in the binding specificity to 6′SLN-LN where binding to other glycan receptors at 40 µg/ml was minimal when compared to mQa88:Q226L at the same concentration. (**Figure 4B**). The Kd' ∼60 pM for 6′SLN-LN binding of mQa88:Q226L/T189A was in the same range as that of mQa88:Q226L. Both these mutant HAs showed extensive staining to apical surface of human tracheal epithelium consistent with their human-receptor binding properties on the glycan array (**Figure 4D**; data for mQa88:Q226L not shown). The third mutant mQa88:T189A increased specificity to 3′SLN-LN and 3′SLN-LN-LN without altering the Kd' (∼30 pM) when compared to that calculated for the wild-type Qa88 HA (**Figure 4C**). Consistent with its glycan array-binding properties, mQa88:T189A also showed extensive staining to human alveolar tissues (**Figure 4D**).

## Discussion

In this study we investigated glycan-receptor binding properties of avian H9N2 HA given that this subtype has been known to infect and cause disease in humans. Using a combination of structural modeling, glycan array and human tissue binding analyses in this study we quantitatively characterized glycan-receptor binding specificity and affinity of wild-type and mutant forms of WF10 and Qa88 HAs. To our knowledge, such a quantitative description of glycan-binding properties of H9 HA has not been reported earlier.

While Qa88 does show measurable binding to human receptors at concentrations higher than 5 µg/ml, its binding to avian receptors is substantially higher. This relative binding preference to avian receptor is typical of what we have observed with avian-adapted HAs including H5 HA [8], [9], [19]. It is important to note that WF10, which is as HA derived from an avian isolate, showed highly preferential binding to human receptors.

Although the Kd' ∼300 pM for WF10 HA binding to human receptor is 5 fold higher than that of 2009 H1N1 HA [7], a reassorted virus with HA and NA from WF10 and other internal genes from a human-adapted H3N2 virus did not show respiratory droplet transmission in ferrets [15]. Repeated passaging of this reassorted virus in ferrets led to a strain (RCP10) that had additional mutations in HA and NA and transmitted via respiratory droplets in ferrets. One of the mutations Thr-189→Ala is in the RBS of H9 HA while the other mutation is in HA2 close to the transmembrane region (unlikely to impact RBS features and hence receptor binding). It was demonstrated that both these mutations are needed for conferring respiratory droplet transmission. Our structural model of WF10 HA–human receptor complex showed that the amino acid in 189 position would indirectly influence glycan-receptor binding through inter-residue interactions. We demonstrated that Thr189→Ala mutation in WF10, mQ88:Q226L and Qa88 HAs increases binding specificity. The fact that the Thr-189→Ala mutation is needed for respiratory droplet transmission highlights the role of improving human receptor specificity in the context of other genes in a reassorted virus in conferring airborne transmissibility. To our knowledge, the effect of the amino acid change at the 189 position on the glycan-binding property of H9 HAs has not been reported earlier.

The contribution of Gln-226 to avian receptor binding and Leu-226 to human receptor binding was corroborated by the preferential avian receptor binding of mWF10:L226Q and human receptor-binding of mQa88:Q226L mutants respectively. Unlike H2 and H3 subtypes where change in receptor binding preference from avian to human receptor has been associated with at least two mutations Q226L and G228S, it appears that in the case of H9 HA a single mutation Q226L might be sufficient to alter its glycan-receptor binding properties. Based on the calculated Kd', the mQa88:Q226L mutant shows 5-fold higher affinity to human receptor than WF10. This suggests that Leu-226 in context of His-156 and Lys-137 in the Qa88 RBS provides a more optimal environment than Leu-226 in the context of Gln-156 and Arg-137 in WF10 RBS for achieving a higher quantitative human receptor affinity. Therefore, this study provides an important framework to understand key mutations such as Gln226→Leu in the context of the entire RBS in different H9 HAs to appropriately monitor the evolution of H9 viruses.

In summary, our results demonstrate that H9 HAs from avian isolates such as WF10 show binding affinity and specificity to human receptors characteristic of HAs from human-adapted subtypes such as H1, H2 and H3. We do note that human adaptation of HA is just one of the many factors that critically govern the adaptation of H9 viruses to human host for efficient airborne human-to-human transmission. While H9N2 subtype is yet to adapt to the human host, reassorted strains with H9 HA and NA have acquired as few as 2 amino acid changes in HA and a single Ile-28→Val change in NA to confer respiratory droplet transmission in ferrets (characteristic trait of human-adapted viruses). Such an outcome has not been possible with HAs from other avian subtypes. The 2 mutations in WF10 HA are much fewer than those reported for H5 HA in the context of reassorted strains that transmit via respiratory droplets between ferrets. Given that natural triple reassortments have led to novel swine-origin H1N1 (2009 H1N1) [1] and H3N2 [2], it is important to monitor H9 HA and NA from strains such as WF10 in the context of their potential natural reassortment with other subtypes. Our study contributes to a framework that facilitates monitoring of molecular changes in RBS of H9 HA that govern its human-receptor binding properties.

## Materials and Methods

### Cloning, baculovirus synthesis, expression and purification of HA

The wild-type WF10 and Qa88 HA genes were synthesized as his-tagged soluble constructs (6×-His tag) as described previously (see Supplementary section of Stevens, J. *et al.* [21] for details). The HA sequences of A/guinea fowl/Hong Kong/WF10/99 (Accession No: AAO46082) and A/quail/Hong Kong/A28945/88(H9N2) (Accession No: AA046081) were taken from Genbank. A synthetic construct was created incorporating AA 19–514 followed by a thrombin cleavage site, a foldon sequence, a 6×-His tag and two stop codons. The construct was codon-optimized for insect cell expression and synthesized by DNA2.0 (Menlo Park, CA). The synthesized gene was sub-cloned into pAcGP67A vector containing gp67 secretion signal. Using pAcGp67-WF10-HA and pAcGp67-Qa88-HA as templates the gene was mutated to yield pAcGp67-L-HA [Gln226Leu], pAcGp67-TL-HA [Thr189Ala, Gln226Leu] and pAcGp67-T-HA [Thr189Ala]. The primers for mutagenesis were designed using PrimerX (http://bioinformatics.org/primerx/) and synthesized by IDT DNA technologies (Coralville, IA). The mutagenesis reaction was carried out using the QuikChange Multi Site-Directed Mutagenesis Kit (Stratagene, CA) *WF10, Qa88, mWF10:T189A, mQa88:T189A, mQa88:Q226L and the double mutant mQa88:T189A/Q226L* baculoviruses were created from their respective plasmids, using Baculogold system (BD Biosciences, CA) as per the manufacturer's instructions. Briefly, recombinant baculoviruses with wild type or mutant forms of WF10 or Qa88 HA gene were used to infect (MOI = 1) suspension cultures of Sf9 cells (Invitrogen, Carlsbad, CA) cultured in BD Baculogold Max-XP SFM (BD Biosciences, San Jose, CA). The infection was monitored and the conditioned media was harvested 3–4 days post-infection. The soluble HA from the harvested conditioned media was purified using Nickel affinity chromatography (HisTrap HP columns, GE Healthcare, Piscataway, NJ). Eluting fractions containing HA were pooled, concentrated and buffer exchanged into 1× PBS pH 8.0 (Gibco) using 100K MWCO spin columns (Millipore, Billerica, MA). The purified protein was quantified using BCA method (Pierce).

### Binding of recombinant WF10, Qa88 and mutant HAs to human tracheal and alveolar tissue sections

Paraffinized human tracheal (US BioChain) tissue sections were deparaffinized, rehydrated and incubated with 1% BSA in PBS for 30 minutes to prevent non-specific binding. HA was pre-complexed with primary antibody (mouse anti 6× His tag, Abcam) and secondary antibody (Alexa fluor 488 goat anti mouse, Invitrogen) in a molar ratio of 4∶2∶1, respectively, for 20 minutes on ice. The tissue binding was performed over different HA concentrations (starting from highest concentration of 40 or 60 µg/ml) by diluting the pre-complexed HA in 1% BSA-PBS. Tissue sections were then incubated with the HA-antibody complexes for 3 hours at RT. The tissue sections were counterstained by propidium iodide (Invitrogen; 1100 in TBST). The tissue sections were mounted and then viewed under a confocal microscope (Zeiss LSM 700 laser scanning confocal microscopy). Sialic-acid specific binding of HAs to tissue sections was confirmed by loss of staining after pre-treatment with Sialidase A (recombinant from *Arthrobacter ureafaciens*, Prozyme), This enzyme has been demonstrated to cleave the terminal Neu5Ac from both Neu5Acα2→3Gal and Neu5Acα2→6Gal motifs. In the case of sialidase pretreatment, tissue sections were incubated with 0.2 units of Sialidase A for 3 hours at 37°C prior to incubation with the proteins. The loss of staining of a representative HAs after sialidase pretreatment is shown in **Figure S1**. On the other hand, absence of pretreating tissue with sialidase showed extensive staining with these HAs (**Figure 1D** **and** **4D**).

### Dose dependent direct binding of WF10, Qa88 and mutant HAs

To investigate the multivalent HA-glycan interactions a streptavidin plate array comprising of representative biotinylated α2→3 and α2→6 sialylated glycans was used as described previously [9]. 3′SLN, 3′SLN-LN, 3′SLN-LN-LN are representative avian receptors. 6′SLN and 6′SLN-LN are representative human receptors (see **Table S1** for glycan structure details). The biotinylated glycans were obtained from the Consortium of Functional Glycomics through their resource request program. Streptavidin-coated High Binding Capacity 384-well plates (Pierce) were loaded to the full capacity of each well by incubating the well with 50 µl of 2.4 µM of biotinylated glycans overnight at 4°C. Excess glycans were removed through extensive washing with PBS at room temperature (∼22°C). The trimeric HA unit comprises of three HA monomers (and hence three RBS, one for each monomer). The spatial arrangement of the biotinylated glycans in the wells of the streptavidin plate array favors binding to only one of the three HA monomers in the trimeric HA unit. Therefore in order to specifically enhance the multivalency in the HA-glycan interactions, the recombinant HA proteins were precomplexed with the primary and secondary antibodies in the molar ratio of 4∶2∶1 (HA: primary: secondary). Controlling the molar ratio of antibodies in the mix facilitated arrangement of 4 trimeric HA units in the complex. The premixing of the HA, primary and secondary antibody leading to the defined multivalent presentation of 4 trimeric units of HA has also been described previously [9], [22]. This presentation permitted comparison between the glycan binding affinities of HAs. A stock solution containing appropriate amounts of His-tagged HA protein (corresponding to highest concentrations of 40 or 60 µg/ml used in the binding assays), primary antibody (Mouse anti 6× His tag IgG) and secondary antibody (HRP conjugated goat anti Mouse IgG (Santacruz Biotechnology) in the ratio 4∶2∶1 was incubated on ice for 20 min. Appropriate amounts of this pre-complexed stock HA (corresponding to highest HA concentrations of 40 or 60 µg/ml used in each assay) were diluted to 250 µl with 1% BSA in PBS. 50 µl of this pre-complexed HA was added to each of the glycan-coated wells and incubated at room temperature for 2 hours followed by the above wash steps. The binding signal was determined based on HRP activity using Amplex Red Peroxidase Assay (Invitrogen, CA) according to the manufacturer's instructions. The experiments were done in triplicate. Minimal binding signals were observed in the negative controls including binding of pre-complexed unit to wells without glycans and binding of the antibodies alone to the wells with glycans. The positive controls for verifying binding signals to human receptors included A/South Carolina/1/18 (H1N1) and A/Albany/6/58 (H2N2) since their binding profiles have been benchmarked previously [8], [9]. The positive control for avian receptor binding signal included A/chicken/Pennsylvania/2004 (H2N2) HA which has also been benchmarked previously [8]. The binding parameters, cooperativity (n) and apparent binding constant (Kd'), for HA-glycan binding were calculated by fitting the average binding signal value (from the triplicate analysis) and the HA concentration to the linearized form of the Hill equation: 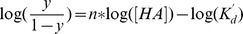
where y is the fractional saturation (average binding signal/maximum observed binding signal).

The linearized Hill equation was plotted for the seven wild type and mutant HAs, WF10, mWF10:T189A, and mWF10:L226Q, Qa88, mQa88:T189A, mQa88:Q226L and mQa88:Q226L/T189A HA were generated to calculate *n* and *K_d_'* (**Figure S2 and S3**). The data points for representative avian (3′SLN-LN) and human (6′SLN-LN) receptors were used for calculations. The linear fit is based on the Hill equation wherein the slope corresponds to the cooperativity factor *n* and the *y* intercept corresponds to -log (*K_d_'*). The *n* value was around 1.3 [9], [19] and the Kd' calculated for concentrations in the range 0.5 µg/ml to 40 µg/ml for all HAs.

### Homology based structural modeling of H9 HAs

Using the SWISS-MODEL web-based automated homology-modeling platform (http://swissmodel.expasy.org/) the structural models of WF10 (GenBank Sequence accession id: AAO46082.1, and Qa88 (GenBank Sequence accession id: AAO46081.1) were constructed. The template structure chosen by SWISS-MODEL was that of crystal structure of A/swine/Hong Kong/9/98 H9N2 HA (PDB ID: 1JSD). The co-crystal structures of A/swine/Hong Kong/9/98 HA with representative avian (PDB ID: 1JSH) and human (PDB ID: 1JSI) receptors were used to build structural models of WF10 and Qa88 in complex with glycan receptors.

## Supporting Information

Figure S1 Sialidase A treated sections of human trachea and alveolus stained with mQa88:Q226L/T189A and mQa88 HA respectively. No binding to either tracheal or alveolar sections is observable. HA (in green) and propidium iodide (in red). On the other hand, absence of pretreating tissue with sialidase showed extensive staining of alveolus with mQa88 HA (**Figure 1D**) and trachea with mQa88:Q226L/T189A HA (**Figure 4D**).
(TIF)

Figure S2 Linearized hill plot for WF10, mWF10:T189A, and mWF10:L226Q HA to calculate *n* and *K_d_'*. Shown are the linearized Hill plot of the WF10 (*A*), mWF10:T189A (*B*), and mWF10:L226Q (*C*) HA-glycan binding data obtained by serial dilution of the precomplexed HA units. The data points for representative avian (3′SLN-LN) and human (6′SLN-LN) receptor are shown. The linear fit is based on the Hill equation (see Materials and Methods), wherein the slope corresponds to the cooperativity factor *n* and the *y* intercept corresponds to -log (*K_d_'*).
(TIF)

Figure S3 Linearized hill plot for Qa88 and mQa88 HA to calculate *n* and *K_d_'*. Shown are the linearized Hill plot of the Qa88 (*A*), mQa88:Q226L (*B*), mQa88:T189A (*C*) and mQa88: Q226l/T189A (D) HA-glycan binding data obtained by serial dilution of the precomplexed HA units. The data points for representative avian (3′SLN-LN) and human (6′SLN-LN) receptor are shown. The linear fit is based on the Hill equation (see Materials and Methods), wherein the slope corresponds to the cooperativity factor *n* and the *y* intercept corresponds to -log (*K_d_'*).
(TIF)

Table S1 Expanded nomenclature of glycans described in the manuscript. Key: Neu5Ac: N-acetyl D-neuraminic acid; Gal: D-galactose; GlcNAc: N-acetyl D-glucosamine. α/β: anomeric configuration of the pyranose sugars. All the sugars used in the glycan array (which do not include LSTa and LSTc) are linked via a spacer to biotin (-Sp-LC-LC-Biotin as described in (http://www.functionalglycomics.org/static/consortium/resources/resourcecored5.shtml).
(PDF)
