# Supplementary figures and images for: Quantitative Characterization of Glycan-Receptor Binding of H9N2 Influenza A Virus Hemagglutinin
Source: PLoS One. 2013 Apr 23;8(4):e59550. doi: 10.1371/journal.pone.0059550 (PMC3634032; doi:10.1371/journal.pone.0059550)

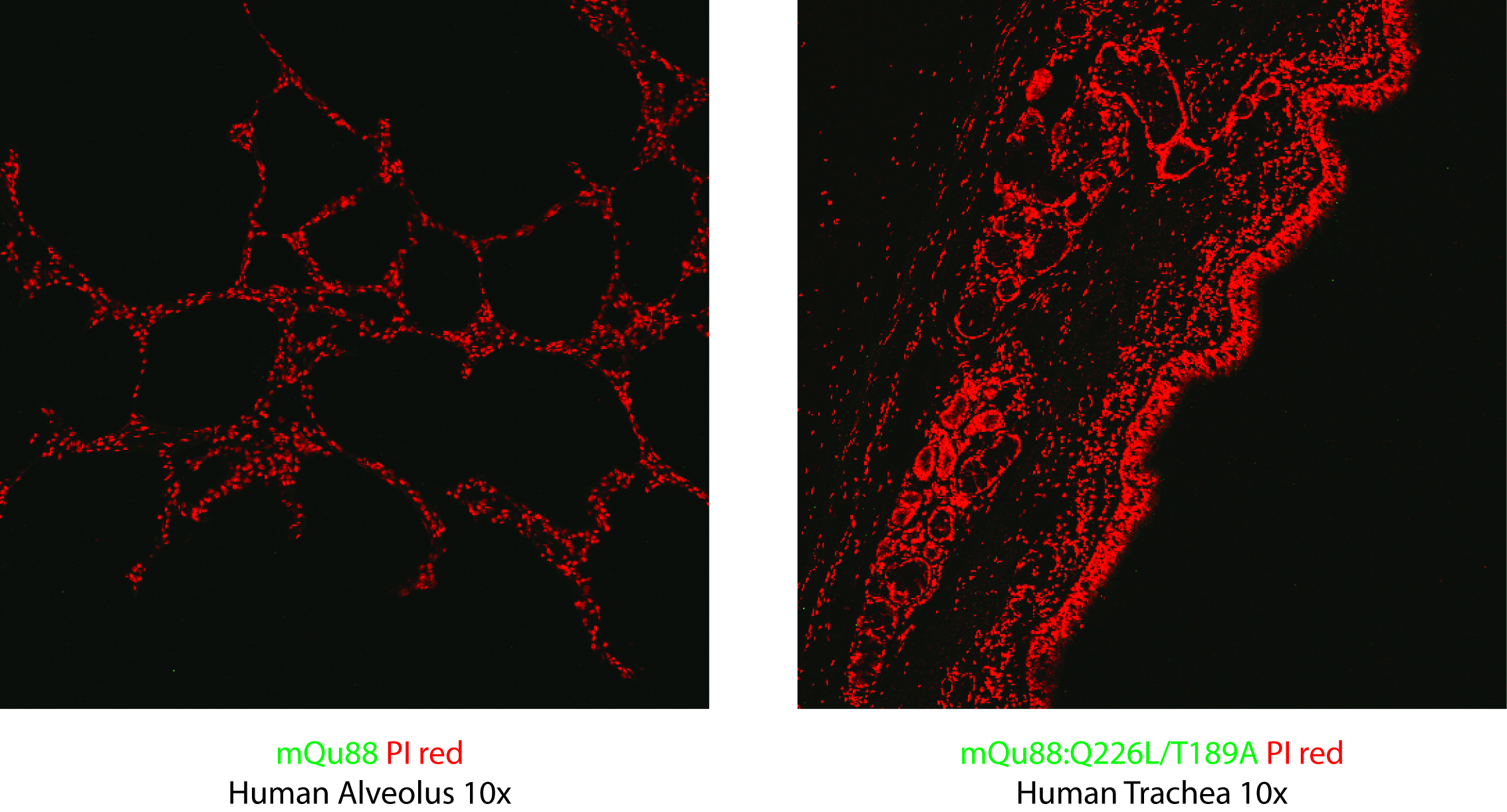

Supplement: Figure S1 — Sialidase A treated sections of human trachea and alveolus stained with mQa88:Q226L/T189A and mQa88 HA respectively. No binding to either tracheal or alveolar sections is observable. HA (in green) and propidium iodide (in red). On the other hand, absence of pretreating tissue with sialidase showed extensive staining of alveolus with mQa88 HA ( Figure 1D ) and trachea with mQa88:Q226L/T189A HA ( Figure 4D ). (TIF) [file pone.0059550.s001.tif]

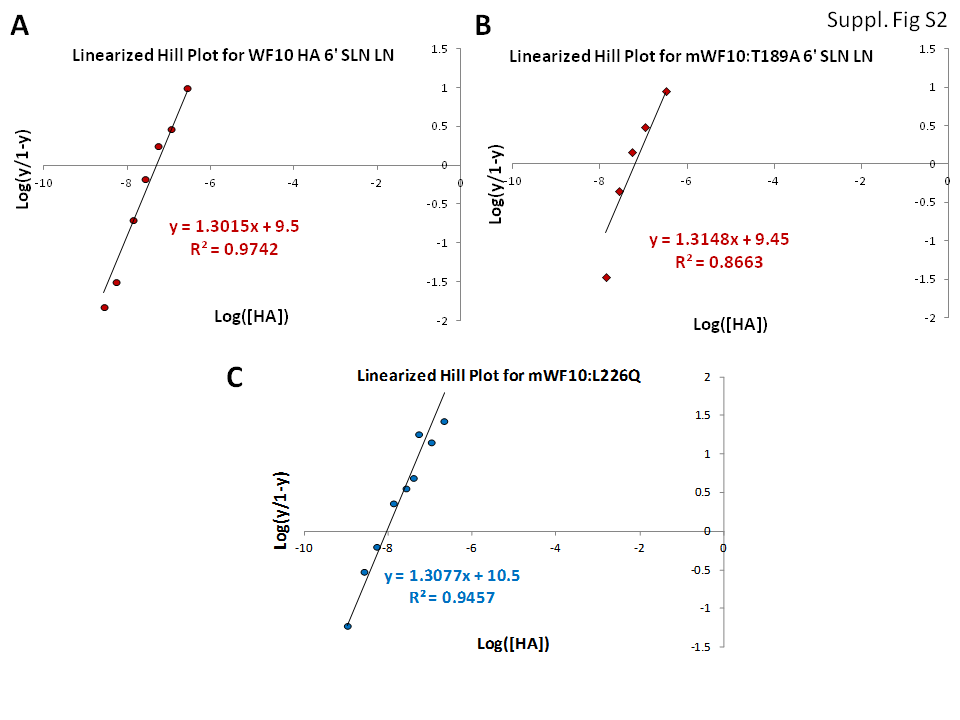

Supplement: Figure S2 — Linearized hill plot for WF10, mWF10:T189A, and mWF10:L226Q HA to calculate n and Kd'. Shown are the linearized Hill plot of the WF10 (A), mWF10:T189A (B), and mWF10:L226Q (C) HA-glycan binding data obtained by serial dilution of the precomplexed HA units. The data points for representative avian (3′SLN-LN) and human (6′SLN-LN) receptor are shown. The linear fit is based on the Hill equation (see Materials and Methods), wherein the slope corresponds to the cooperativity factor n and the y intercept corresponds to -log (Kd'). (TIF) [file pone.0059550.s002.tif]

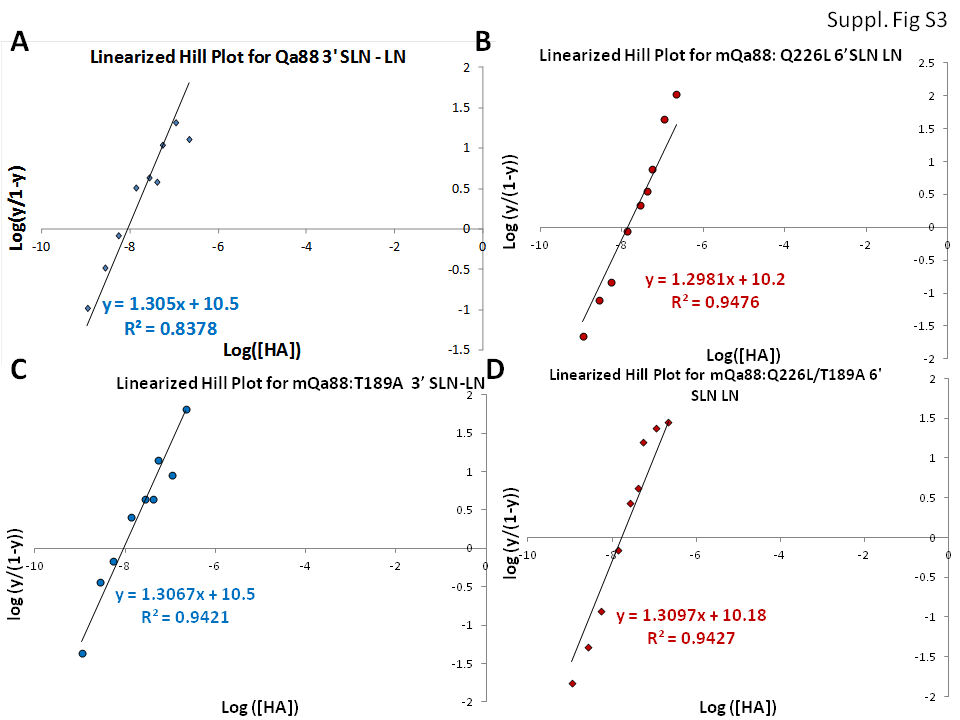

Supplement: Figure S3 — Linearized hill plot for Qa88 and mQa88 HA to calculate n and Kd'. Shown are the linearized Hill plot of the Qa88 (A), mQa88:Q226L (B), mQa88:T189A (C) and mQa88: Q226l/T189A (D) HA-glycan binding data obtained by serial dilution of the precomplexed HA units. The data points for representative avian (3′SLN-LN) and human (6′SLN-LN) receptor are shown. The linear fit is based on the Hill equation (see Materials and Methods), wherein the slope corresponds to the cooperativity factor n and the y intercept corresponds to -log (Kd'). (TIF) [file pone.0059550.s003.tif]
