# Supplementary material for: Quantitative Characterization of Glycan-Receptor Binding of H9N2 Influenza A Virus Hemagglutinin
Source: PLoS One. 2013 Apr 23;8(4):e59550. doi: 10.1371/journal.pone.0059550 (PMC3634032; doi:10.1371/journal.pone.0059550)
Supplement: Table S1 — Expanded nomenclature of glycans described in the manuscript. Key: Neu5Ac: N-acetyl D-neuraminic acid; Gal: D-galactose; GlcNAc: N-acetyl D-glucosamine. α/β: anomeric configuration of the pyranose sugars. All the sugars used in the glycan array (which do not include LSTa and LSTc) are linked via a spacer to biotin (-Sp-LC-LC-Biotin as described in (http://www.functionalglycomics.org/static/consortium/resources/resourcecored5.shtml). (PDF) [file pone.0059550.s004.pdf]

Supplementary Table S1. Expanded nomenclature of glycans described in the manuscript

**Glycan**	**Expanded nomenclature**
3'SLN	Neu5Acα2-3Galβ1-4GlcNAcβ1-
6'SLN	Neu5Acα2-6Galβ1-4GlcNAcβ1-
3'SLN-LN	Neu5Acα2-3Galβ1-4GlcNAcβ1-3Galβ1-4GlcNAcβ1-
LSTa	Neu5Acα2-3Galβ1-3GlcNAcβ1-3Galβ1-4Glcβ1-
6'SLN-LN	Neu5Acα2-6Galβ1-4GlcNAcβ1-3Galβ1-4GlcNAcβ1-
LSTc	Neu5Acα2-6Galβ1-4GlcNAcβ1-3Galβ1-4Glcβ1-
3'SLN-LN-LN	Neu5Acα2-3Galβ1-4GlcNAcβ1-3Galβ1-4GlcNAcβ1-3Galβ1-4GlcNAcβ1-

Key: Neu5Ac: N-acetyl D-neuraminic acid; Gal: D-galactose; GlcNAc: N-acetyl D-glucosamine. α / β: anomeric configuration of the pyranose sugars. All the sugars used in the glycan array (which do not include LSTa and LSTc) are linked via a spacer to biotin (-Sp-LC-LC-Biotin as described in (http://www.functionalglycomics.org/static/consortium/resources/resourcecored5.shtml)
